# A Simple, Highly Sensitive, and Highly Specific Dot-Blot-Based Immunoassay for Serodiagnosis of HTLV-1 in Resource-Limited Settings

**DOI:** 10.3390/tropicalmed10100279

**Published:** 2025-09-26

**Authors:** Haohan Zhuang, Shanhai Ou, Lixing Wang, Hongzhi Gao

**Affiliations:** 1 Central Laboratory, The Second Affiliated Hospital of Fujian Medical University, Quanzhou 362000, China; zhuanghaohan@163.com (H.Z.); gnixil@fjmu.edu.cn (L.W.); 2 Fujian Key Laboratory of Lung Stem Cells, The Second Affiliated Hospital of Fujian Medical University, Quanzhou 362000, China; 3 Xiamen Blood Center, Xiamen 361000, China; oushanhai2025@163.com

**Keywords:** human T-cell leukemia virus type 1, dot-blot, serodiagnosis, resource-limited settings

## Abstract

Human T-cell leukemia virus type 1 (HTLV-1), the first identified human retrovirus, is associated with adult T-cell leukemia (ATL) and HTLV-1-associated myelopathy/tropical spastic paraparesis (HAM/TSP). The lack of effective antiviral therapies or vaccines highlights the critical importance of early diagnosis in managing HTLV-1-associated diseases. However, current commercial immunoassays, including enzyme immunoassays, line immunoassays, particle agglutination tests, and Western blots, are often limited by the need for specialized equipment and high costs, which restrict their accessibility in resource-poor regions. To address these challenges, we developed a novel dot-blot immunoassay using HTLV-1 P19 and GP46 synthetic peptides in combination with a precipitating tetramethylbenzidine (TMB) substrate. This innovative approach enables instrument-free visual detection through the formation of distinct blue-brown precipitates. Validation of this immunoassay with 179 clinical serum samples demonstrated 100% specificity and 91% sensitivity. Our assay offers a simple, cost-effective, and field-applicable diagnostic solution for HTLV-1 screening in resource-limited settings, potentially enhancing global surveillance of this neglected pathogen.

## 1. Introduction

Human T-cell leukemia virus type 1 (HTLV-1), the first identified human retrovirus (discovered in 1980) [1], infects over 10 million individuals worldwide [2]. HTLV-1 infection imposes a lifelong risk of adult T-cell leukemia (ATL), a highly aggressive T-cell malignancy, and HTLV-1-associated myelopathy/tropical spastic paraparesis (HAM/TSP), a chronic progressive inflammatory disorder of the central nervous system [3].

Compared with the well-characterized retrovirus human immunodeficiency virus (HIV-1), HTLV-1 has received relatively less research attention. Though the two share similar transmission routes (vertical transmission, sexual contact, bloodborne exposure) [4], HTLV-1 shows more geographically restricted distribution (endemic in Japan, the Caribbean, and sub-Saharan Africa) versus HIV-1’s global pandemic [2,5]. Pathogenically, HTLV-1 establishes persistent infection via clonal proliferation of infected CD4+ T cells (with low viral load and stable genetic structure), while HIV-1 causes progressive immunodeficiency through continuous replication and rapid genetic evolution. Clinically, this translates to HTLV-1’s long latency (only 2–5% of carriers develop severe manifestations) versus the near-invariable progression of untreated HIV-1 infection to acquired immunodeficiency syndrome (AIDS) [6,7]. Notably, HIV-1 has highly effective therapeutic strategies, whereas HTLV-1 treatments mostly adopt HIV-1-derived interventions (due to a lack of standardized protocols/guidelines). Despite decades of research, HTLV-1 still has no prophylactic vaccines or curative agents, highlighting the importance of early diagnosis for transmission control [8].

Current HTLV-1 diagnosis employs a stepwise algorithm: initial screening (typically enzyme immunoassay [EIA]), followed by confirmatory testing (e.g., Western blots [WBs], line immunoassays [INNO-LIAs], or molecular techniques, such as polymerase chain reaction [PCR]) [9,10]. While reliable, these methods require sophisticated instrumentation, specialized training, and substantial financial resources, constraining their utility in resource-limited settings. To address the diagnostic gap in HTLV-1 detection, we developed a cost-effective dot-blot assay using synthetic HTLV-1 P19 and GP46 peptides as antigens. The assay produces distinct blue-brown precipitates at antibody binding sites via a precipitating tetramethylbenzidine (TMB) substrate, enabling instrument-free visual interpretation. This approach eliminates the need for the complex infrastructure required by conventional methods such as EIA, WB, and PCR. By reducing costs and enhancing accessibility, it provides a practical solution for resource-limited settings to improve HTLV-1 surveillance and epidemiological investigations.

## 2. Methods

### 2.1. Clinical Samples

A total of 78 HTLV-1-positive human serum samples and 101 HTLV-1-negative control sera were obtained from Xiamen Blood Center (Xiamen, China). All positive sera were initially screened at Xiamen Blood Center using commercial ELISA kits, including Wantai HTLV-1/2 antibody ELISA kits (Wantai BioPharm, Beijing, China) and Murex HTLV I + II kits (Diasorin S.p.A., Dartford, UK). Subsequently, the positive results were confirmed by Western blot analysis using an MP Biomedicals HTLV Blot 2.4 kit (MP Biomedicals, Santa Ana, CA, USA). HTLV-1-negative sera were defined as samples that yielded non-reactive results in HTLV-1 ELISA assays. Additionally, 10 serum samples from hepatitis B virus (HBV)-positive blood donors and 10 from hepatitis C virus (HCV)-positive blood donors were also acquired from the same center, and these samples were used to evaluate the cross-reactivity of the dot-blot assay established in this study. All blood donors provided written informed consent prior to blood donation and completed relevant questionnaires at the Xiamen Blood Center. Their information was kept confidential through strict measures.

### 2.2. Reagents and Materials

The 0.22 μm nitrocellulose membrane (Cat. No. YA1710) was purchased from Solarbio Life Sciences (Beijing, China). Skim milk (Cat. No. FD0080), horseradish peroxidase (HRP)-conjugated goat anti-human IgG secondary antibody (Cat. No. FD0125), and the enhanced chemiluminescence (ECL) substrate for exposure (Cat. No. FD8020) were obtained from Fude Biological Co., Ltd. (Hangzhou, China). The precipitating 3,3′,5,5′-tetramethylbenzidine (TMB) color development solution (Cat. No. PH0410) was supplied by Phygene Biotechnology Co., Ltd. (Fuzhou, China).

### 2.3. Dot-Blot Assay

Two peptides derived from the HTLV-1 Gag protein P19 (amino acid sequence: CQIPPPYVEPTAPQVL) and surface envelope glycoprotein (Env) GP46 (amino acid sequence: CFLNTEPSQLPPTAPPLLPHSNLDHI) were synthesized by Sangon Biotech Co., Ltd. (Shanghai, China). Both peptides had a purity of >98%, confirmed by high-performance liquid chromatography (HPLC) purification and mass spectrometry analysis. Each peptide was aliquoted into five vials, with 1 mg of peptide per vial. A 100 μL volume of dimethyl sulfoxide (DMSO) was added to each vial to completely dissolve the lyophilized peptide. Subsequently, 900 μL of phosphate-buffered saline (PBS) was added to prepare a primary working solution at a concentration of 1 mg/mL. Serial dilutions were then performed using a 10% (*v*/*v*) DMSO-PBS solution to generate secondary working solutions at 0.1 mg/mL and 0.01 mg/mL. The 0.22 μm nitrocellulose (NC) membrane was precut into rectangular pieces (1.5 cm × 2.5 cm). Using a micropipette (Eppendorf, Hamburg, Germany), 1 μL of each peptide solution (1, 0.1, and 0.01 mg/mL) was spotted onto separate membrane pieces and allowed to air-dry at room temperature (RT) for 15 min to immobilize the peptides. Subsequently, the membranes were blocked with 5 mL of blocking buffer (5% skim milk in PBS containing 0.05% Tween 20 [PBST]) for 30 min on a shaker at gentle speed. The blocked membranes were then incubated in a serum solution, which consisted of 100 μL of HTLV-1-positive, HTLV-1-negative, hepatitis B virus (HBV)-positive, or hepatitis C virus (HCV)-positive serum diluted into 5 mL of blocking buffer, for 1 h at RT with gentle agitation. After incubation, the membranes were washed three times with PBST, with each washing lasting 5 min. Then, the membranes were incubated in a secondary antibody solution, which was prepared by diluting the horseradish peroxidase (HRP)-conjugated goat anti-human IgG secondary antibody at a 1:5000 dilution in the blocking buffer, for 1 h at RT with gentle agitation. Following this, the membranes were washed three times with PBST for 5 min each. To validate the feasibility of using P19 and GP46 peptides as detection agents in the dot-blot assay, membranes incubated with 12 HTLV-1-positive and 1 HTLV-1-negative serum samples were incubated with the ECL substrate, and the dot signals were detected by exposure using a chemiluminescence imager (Invitrogen; Thermo Fisher Scientific, Inc., Waltham, MA, USA). Subsequently, the remaining membranes, separately incubated with 66 HTLV-1-positive, 100 HTLV-1-negative, 10 HBV-positive, and 10 HCV-positive serum samples, were immersed in the precipitating tetramethylbenzidine (TMB) solution for 90 s, and the developed dots were observed visually. Note that the dissolved peptide solution must be freshly prepared and used immediately; peptide-immobilized NC membranes should not be stored and must be used for detection on the same day of preparation to avoid signal loss due to peptide degradation or membrane damage.

### 2.4. Quantification of Dot-Blot Spot Intensity

To quantify the spot intensity in dot-blot assays for determining the positive or negative status of experimental results, ImageJ Win64 software (National Institutes of Health, Bethesda, MD, USA) was employed. Raw dot-blot images were first processed through two sequential commands in the software: the “Edit > Invert” command to invert the image grayscale and the “Process > Subtract Background” command to eliminate background noise, ensuring accurate detection of target spots. After preprocessing, the target spots of interest were manually outlined using the selection tool in ImageJ, and the “Analyze > Measure” command was executed to acquire the mean gray value of each outlined spot (serving as the quantitative indicator of spot intensity). Based on the visual color development intensity of the spots, the spot signals were categorized into four grades: strong (intense color development), moderate, weak, and no signal. Ten representative spots were selected for each grade to measure their mean gray values, and the average mean gray values were calculated as approximately 70.184 (strong), 40.596 (moderate), 1.194 (weak), and 0.214 (no signal). Finally, a cutoff value of 1.10 for spot signals was established based on these average values, with spots having a mean gray value exceeding 1.10 defined as positive and those below 1.10 defined as negative.

### 2.5. Determination of the Limit of Detection (LoD) of the Assay

To determine the limit of detection (LoD) of the present assay, two HTLV-1-positive patient serum samples were selected. Among them, Sample No. 13 (HTLV-1-positive) was a strong-signal serum, and Sample No. 15 (HTLV-1-positive) was a moderate-signal serum. The experimental procedure was performed in accordance with the description provided in Section 2.3 (Dot-Blot Assay). For each serum sample, four dilution levels were set up, including the initial working solution (100 μL of HTLV-1-positive serum diluted in 5 mL of blocking buffer), 10-fold dilution, 100-fold dilution, and 1000-fold dilution. Finally, the results were developed and visualized using a precipitating TMB solution.

### 2.6. Assessment of Assay Sensitivity and Specificity

To evaluate the performance of the proposed dot-blot assay, the sensitivity and specificity were calculated as follows: the sensitivity was determined by analyzing HTLV-1-positive sera (confirmed by ELISA and Western blots) from Xiamen Blood Center. Samples showing a positive signal for either P19 or GP46, or both peptides, were classified as true positives (TP), while those with no detectable signal were classified as false negatives (FN). The sensitivity was computed using the following formula:

sensitivity = TP/(TP + FN).

The specificity was assessed using HTLV-1-negative sera from Xiamen Blood Center. Samples exhibiting a positive signal for either P19 or GP46, or both peptides, were classified as false positives (FP), whereas those with no signal were classified as true negatives (TN). The specificity was calculated using the following formula:

specificity = TN/(TN + FP).

### 2.7. Alignment of P19 and GP46 Peptide Sequences

The P19 and GP46 peptides were subjected to BLAST analysis using the MegAlign v7.1.0 software (DNASTAR Lasergene). The amino acid sequences of these peptides were compared against entries from the UniProt and NCBI databases. Specifically, the P19 peptide was aligned with the following sequences: P03362 (Human T-cell leukemia virus 1, strain Japan ATK-1, subtype A), P0C211 (Human T-cell leukemia virus 1, isolate Melanesia mel5, subtype C), P14078 (Human T-cell leukemia virus 1, isolate Caribbea HS-35, subtype A), and GenBank: XTJ61547.1 (Simian T-lymphotropic virus 1). The GP46 peptide was aligned with P03381 (Human T-cell leukemia virus 1, strain Japan ATK-1, subtype A), P0C212 (Human T-cell leukemia virus 1, isolate Melanesia mel5, subtype C), P14075 (Human T-cell leukemia virus 1, isolate Caribbean HS-35, subtype A), and GenBank: ASU50907.1 (Simian T-lymphotropic virus 1). The alignments were performed using the Clustal W method in MegAlign, and residues that matched the consensus sequence exactly were indicated by solid black shading.

## 3. Results

### 3.1. Dot-Blot Results Obtained Using ECL Detection

**Figure 1:**
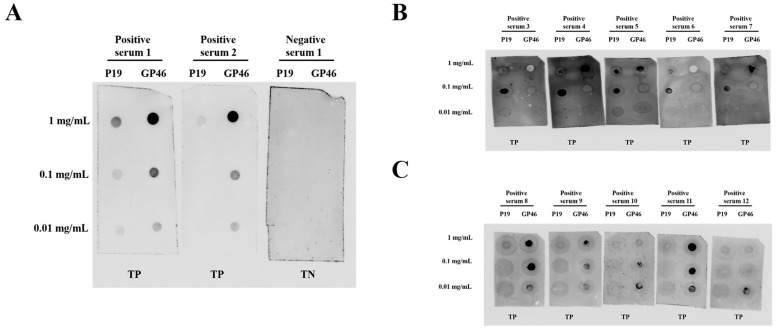
Dot-blot results for P19 and GP46 peptides using ECL exposure for twelve HTLV-1-positive sera and one HTLV-1-negative serum. (**A**): ECL exposure for two HTLV-1-positive sera (labeled 1–2) and one HTLV-1-negative serum (labeled 1). (**B**): ECL exposure for five HTLV-1-positive sera (labeled 3–7). (**C**): ECL exposure for five HTLV-1-positive sera (labeled 8–12). Note: TP: true-positive; TN: true-negative.

To assess the potential of the designed P19 and GP46 peptides as antigens for detecting HTLV-1-specific antibodies in a dot-blot assay, we conducted an experiment using electrochemiluminescence (ECL) detection. Twelve randomly selected HTLV-1-positive patient sera and one negative patient serum were tested. As illustrated in Figure 1, the positive sera (labeled 1–12) demonstrated strong recognition of both P19 and GP46 peptides. The ECL signals exhibited a clear concentration-dependent decrease in intensity as the peptide concentration was reduced from 1 mg/mL to 0.01 mg/mL, thereby confirming true-positive (TP) results. In contrast, the negative serum (labeled 1) showed no detectable signal, with a clean background, confirming a true-negative (TN) outcome. These results provide compelling evidence supporting the feasibility of using P19 and GP46 peptides for the detection of HTLV-1-positive sera via dot-blot assays.

### 3.2. Dot-Blot Results Obtained Using TMB Detection

**Figure 2:**
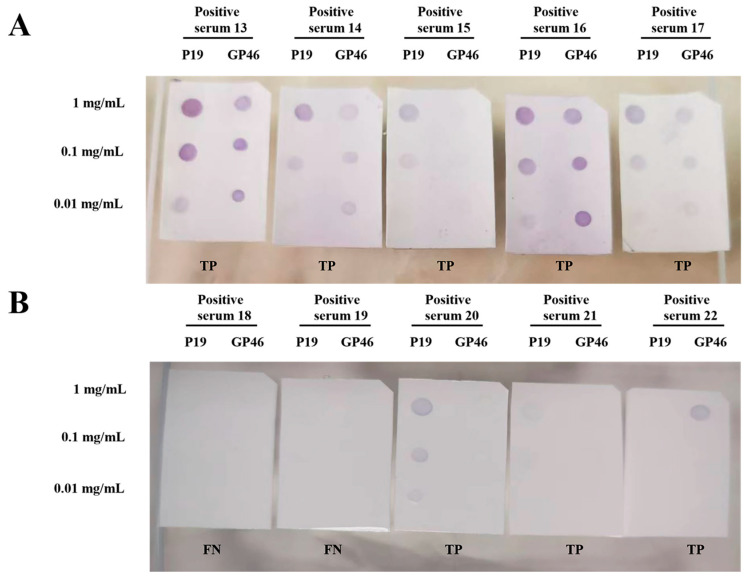
Dot-blot results for P19 and GP46 peptides using TMB detection for ten HTLV-1-positive sera. (**A**): TMB detection for five HTLV-1-positive sera (labeled 13–17). (**B**): TMB detection for five HTLV-1-positive sera (labeled 18–22). Note: TP: true-positive; FN: false-negative.

Following the successful ECL detection of P19 and GP46 peptides in the initial 12 positive samples, the remaining 66 HTLV-1-positive and 100 HTLV-1-negative sera were analyzed using the precipitating tetramethylbenzidine (TMB) solution. By reacting with the horseradish peroxidase (HRP) groups conjugated to the secondary antibodies, the precipitating TMB solution was expected to form visible blue-brown spots on nitrocellulose membranes, thereby eliminating the need for an exposure instrument required in the conventional ECL exposure process. As illustrated in Figure 2A, the positive sera labeled 13–17 developed distinct blue-brown spots within 90 s of TMB staining, indicative of true-positive results. Similar to the ECL findings, the signal intensity showed a concentration-dependent attenuation. Figure 2B presents the results for another five positive sera (labeled 18–22), among which samples 18 and 19 failed to produce any detectable signal, thus being classified as false negatives (FN). The complete precipitating TMB detection results for the remaining 56 positive sera (labeled 23–78) and 100 negative sera (labeled 2–101) are available in the Supplementary Materials (Supplementary Figures S1 and S2).

### 3.3. Assessment of Cross-Reactivity and Limit of Detection (LoD) of Dot-Blot Assay

To verify the cross-reactivity of the present assay, we obtained HBV- and HCV-positive patient serum samples from our available resources, with 10 serum samples for each virus. The results showed that these sera did not react with the P19 and GP46 peptides designed in this study, and the negative results are presented in Supplementary Figure S3.

To determine the LoD of the present assay, two HTLV-1-positive patient serum samples with different signal intensities (Sample Nos. 13 and 15) were chosen. The results (Supplementary Figure S4) showed that Sample No. 13 (strong-signal positive serum) still exhibited detectable positive signals even after 100-fold dilution; in contrast, Sample No. 15 (moderate-signal positive serum) only showed weak positive signals after 10-fold dilution. Considering that in this assay, 100 μL of undiluted serum was added to the blocking buffer when using HTLV-1-positive serum, and the sera were collected between 2016 and 2018 (during which the antibodies may have undergone partial degradation due to storage), the LoD of this assay is conservatively estimated to be within the dilution range of 1:10 to 1:100.

### 3.4. Assessment of Assay Sensitivity and Specificity

To evaluate the performance of the proposed dot-blot assay, the sensitivity and specificity were calculated using the following formulas: Sensitivity = TP/(TP + FN) and Specificity = TN/(TN + FP). A total of 78 HTLV-1-positive and 101 HTLV-1-negative serum samples were tested using both ECL and TMB detection methods. Among the positive samples, 71 were correctly identified as true positives (TP), while 7 were misclassified as false negatives (FN), yielding a sensitivity of 91% (71/78). Meanwhile, all 101 negative samples tested negative, yielding 101 true negatives (TN) with no false-positives (FP), thereby achieving a specificity of 100% (101/101). Overall, the dot-blot assay demonstrated a sensitivity of 91% and a specificity of 100% under the tested conditions.

### 3.5. Peptide Sequence Alignment Result

**Figure 3:**
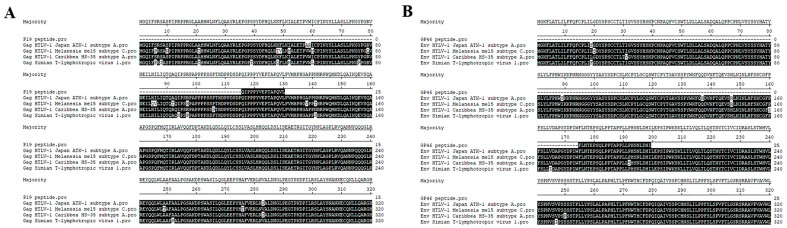
Sequence alignment of the P19 and GP46 peptides against selected proteins from UniProt and NCBI databases. (**A**): Sequence alignment of the P19 peptide against UniProt P03362 (Human T-cell leukemia virus 1, strain Japan ATK-1, subtype A), P0C211 (Human T-cell leukemia virus 1, isolate Melanesia mel5, subtype C), P14078 (Human T-cell leukemia virus 1, isolate Caribbea HS-35, subtype A), and NCBI GenBank: XTJ61547.1 (Simian T-lymphotropic virus 1). (**B**): Sequence alignment of the gp46 peptide against UniProt P03381 (Human T-cell leukemia virus 1, strain Japan ATK-1, subtype A), P0C212 (Human T-cell leukemia virus 1, isolate Melanesia mel5, subtype C), P14075 (Human T-cell leukemia virus 1, isolate Caribbea HS-35, subtype A), and NCBI GenBank: ASU50907.1 (Simian T-lymphotropic virus 1).

To assess the broad applicability of the P19 and GP46 peptides designed in this study, we performed sequence alignments of these peptides against the UniProt and NCBI databases. The alignment results indicated that both peptides showed high homology with several strains of human T-cell leukemia virus type 1 (HTLV-1), including strain Japan ATK-1 (subtype A), isolate Caribbea HS-35 (subtype A), isolate Melanesia mel5 (subtype C), and simian T-lymphotropic virus type 1 (STLV-1). Subsequently, we downloaded the highly homologous protein sequences and conducted further alignments using the MegAlign V7.1.0 software. As illustrated in Figure 3, the P19 peptide demonstrated 100% homology with all the selected protein sequences. The GP46 peptide was also 100% homologous with three of the sequences, and it had only one amino acid difference with the isolate Caribbea HS-35 (subtype A) of HTLV-1. These findings suggest that the method developed in this study has the potential to be extended for the detection of simian T-lymphotropic virus type 1 and various HTLV-1 strains in other endemic regions.

## 4. Discussion

The widely adopted confirmatory serologic diagnostic methods for HTLV-1 infection include the Western blot (WB) assay developed by MP Diagnostics, and the INNO-LIA™ immunoblot system (Fujirebio, Tokyo, Japan) [10]. Both assays rely on HTLV-1-specific antigens, such as GAG proteins (P19 and P24) and ENV glycoproteins (GP46 and P21), to detect virus-specific antibodies in patient sera [9]. However, these methods may not be cost-effective or readily accessible in resource-limited settings. To address these challenges, this study employed synthetic peptides corresponding to P19 and GP46 as antigenic substrates for dot-blot analysis, aiming to optimize cost-efficiency while maintaining diagnostic specificity in the Chinese epidemiological context. This approach was informed by previous reports of the cross-reactivity of P21 with antibodies generated during Plasmodium infection [11], as well as frequent false-positive results associated with P24 [12]. The predominant circulating strain of HTLV-1 in mainland China belongs to the Transcontinental subtype of genotype A, which shares identical sequences with the Transcontinental subtype of HTLV-1 reported in Japan [13]. Previous studies from Japan have demonstrated that P19 and GP46 exhibit excellent specificity and sensitivity for HTLV-1 detection [14]. Consistent with these findings, the sequence alignment of the P19 and GP46 peptides used in this study against databases (UniProt and NCBI) revealed high homology with HTLV-1 Caribbean subtype A, Melanesia subtype C, and the simian T-lymphotropic virus 1 (STLV-1) protein sequences in non-human primates. This high degree of homology suggests that the developed method has the potential to be extended for the detection of simian T-lymphotropic virus type 1 and various HTLV-1 strains in other endemic regions.

The HTLV-1-positive serum samples used in this study, collected between 2016 and 2018, had already undergone multiple rounds of HTLV-1 serological screening at the Xiamen Blood Center, resulting in a very limited quantity available for our research. Additionally, as China is not an endemic region for HTLV-1 infection, obtaining HTLV-1-positive serum samples is particularly challenging. These constraints significantly limited our ability to conduct repeated experiments within the scope of this study. Despite these limitations, our dot-blot assay demonstrated high sensitivity and specificity, providing promising preliminary results. We encourage researchers in HTLV-1-endemic regions to conduct similar experiments to corroborate our findings and assess the reliability of our method in a broader context. Our dot-blot analysis of 78 confirmed HTLV-1-positive serum samples revealed distinct serological recognition patterns: 37 samples (47.4%) exhibited simultaneous reactivity to both P19 and GP46 synthetic peptide antigens, 16 samples (20.5%) exhibited exclusive reactivity to P19, 18 samples (23.1%) reacted solely to GP46, and 7 samples (9.0%) displayed no antigen-specific recognition. The observed mono-reactivity and non-reactivity patterns may be attributed to two principal limitations: prolonged serum storage (archived from 2016 to 2018) potentially inducing antibody degradation and reduced immunoglobulin stability, and insufficient conformational epitope preservation in the 15-amino-acid synthetic peptides (P19), which may fail to sustain optimal antibody–antigen interactions. To enhance diagnostic sensitivity, future refinements may prioritize the use of recombinant P19 and GP46 proteins to improve epitope presentation and mitigate potential false-negative outcomes through multi-antigen verification. The dot-blot analysis could serve as a primary screening test, followed by confirmatory tests such as Western blots (WBs), INNO-LIA, or PCR for definitive detection of HTLV-1 infection.

In recent years, numerous innovative technologies for HTLV-1 detection have been reported. For instance, the Multi-HTLV technique enables high-throughput screening in 96-well plates by designing specific antigens for HTLV-1 and HTLV-2, allowing simultaneous differentiation between the two viruses [15]. The ASSURE HTLV-I/-II Rapid Test developed by Biomedicals Asia Pacific Pte Ltd. completes detection within 15 min and is compatible with whole blood specimens, making it suitable for rapid HTLV infection identification in scenarios such as organ transplantation [16]. Additionally, newly developed duplex real-time PCR can be used to determine the proviral load to assess the emergence of HTLV-1-associated diseases [17]. While these methods offer advantages in specific contexts, they may be limited by high reagent costs or reliance on sophisticated instruments, which may not be feasible in resource-limited settings. In China, the HTLV-1 distribution is highly concentrated, with a recent nationwide screening in blood banks revealing an infection rate of approximately 36.2 per 100,000 individuals in Fujian Province, compared to 2.4 per 100,000 in other provinces [18]. Within Fujian Province, the distribution is uneven, with the highest rate of 171.3 per 100,000 in Ningde City [13]. Globally, many underdeveloped regions with concentrated HTLV-1 distribution, such as Mashhad in northeast Iran [19], the southern Andes of Peru [20], and Atakora in northern Benin [21], may face similar challenges in implementing large-scale serological screening due to fiscal and public health constraints. Given that blocking the vertical transmission of HTLV-1 can effectively reduce disease spread, implementing HTLV-1 screening for pregnant women appears more suitable for these areas [22,23]. The dot-blot method developed in this study provides a cost-effective alternative for HTLV-1 detection, with visible blue-brown spots serving as clear and interpretable results. This approach requires only basic laboratory equipment, such as an analytical balance, a basic shaker, and a micropipette, making it highly accessible. While it may not be ideal for large-scale screening, its simplicity and affordability render it particularly suitable for sporadic HTLV-1 sample detection in resource-limited regions, for instance, in the screening of serum samples from pregnant women in regions with a high HTLV-1 prevalence rate. In this capacity, it has the potential to contribute to global serological and epidemiological investigations of HTLV-1.

## Data Availability

The datasets used and analyzed during the current study are available from the corresponding author upon reasonable request.

## Supplementary Materials

The following supporting information can be downloaded at: https://www.mdpi.com/article/10.3390/tropicalmed10100279/s1, Figure S1: Dot-blot results for P19 and GP46 peptides using TMB detection for 56 HTLV-1-positive sera (labeled 23–78). Note: TP: true-positive; FN: false-negative; Figure S2: Dot-blot results for P19 and GP46 peptides using TMB detection for 100 HTLV-1-negative sera (labeled 2–101); Figure S3: Dot-blot results for P19 and GP46 peptides using TMB detection for 10 hepatitis B virus (HBV)-positive sera and 10 hepatitis C virus (HCV)-positive sera; Figure S4: Dot-blot results for P19 and GP46 peptides using TMB detection for HTLV-1-postive sera 13 and 15 at four dilution levels (initial working solution, 10-fold dilution, 100-fold dilution, and 1000-fold dilution).
