# Supplementary material for: A Simple, Highly Sensitive, and Highly Specific Dot-Blot-Based Immunoassay for Serodiagnosis of HTLV-1 in Resource-Limited Settings
Source: Trop Med Infect Dis. 2025 Sep 26;10(10):279. doi: 10.3390/tropicalmed10100279 (PMC12567712; doi:10.3390/tropicalmed10100279)
Supplement: Supplementary file 1 [file tropicalmed-10-00279-s001.zip › tropicalmed-3779445-supplementary.pdf]

Supplementary Materials

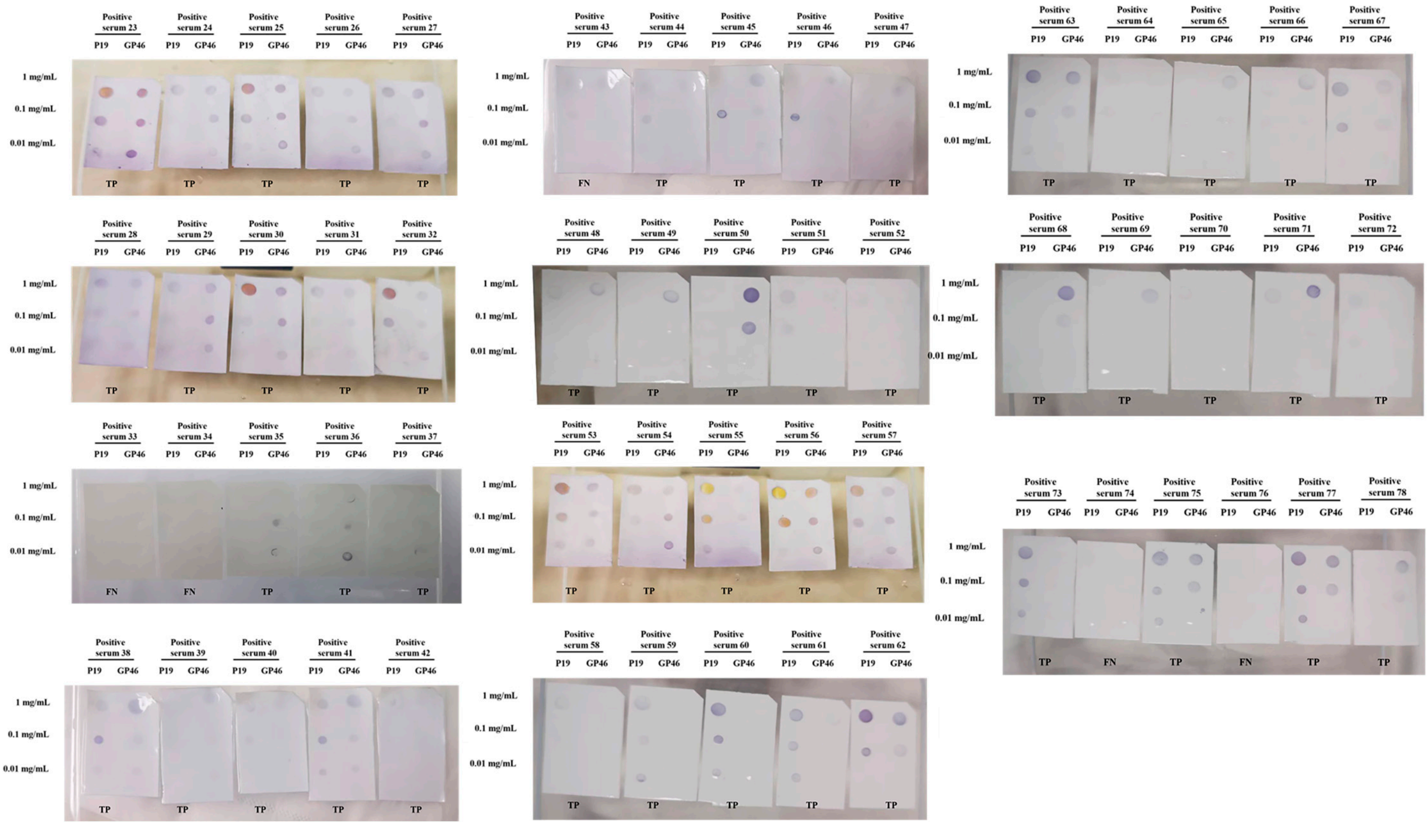

Figure S1. Dot-blot results for P19 and GP46 peptides using TMB detection for 56 HTLV-1-positive sera (labeled 23–78). Note: TP: true-positive; FN: false-negative.

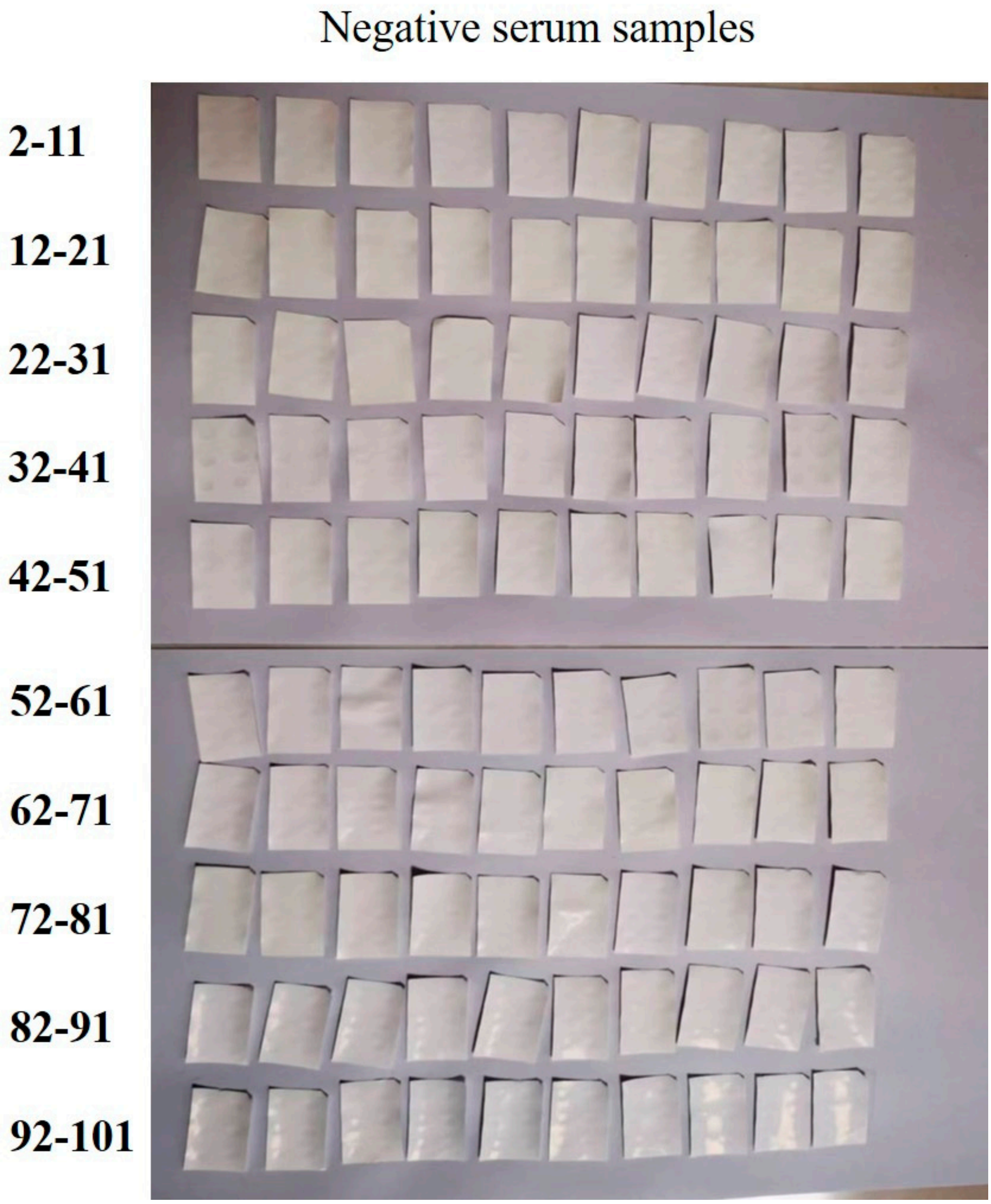

Figure S2. Dot-blot results for P19 and GP46 peptides using TMB detection for 100 HTLV-1-negative sera (labeled 2–101).

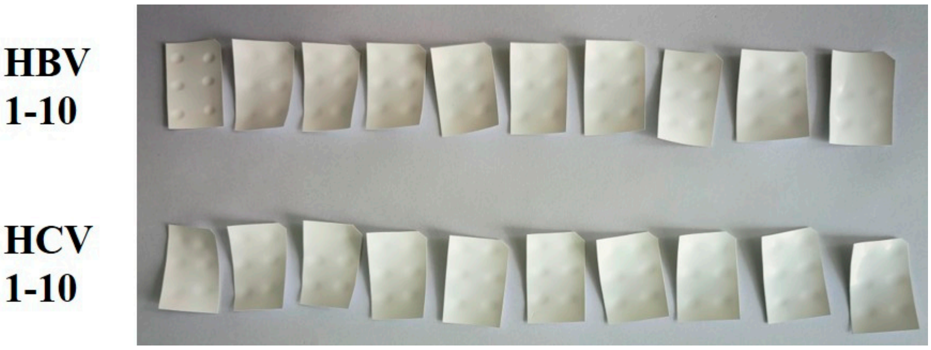

Figure S3. Dot-blot results for P19 and GP46 peptides using TMB detection for 10 hepatitis B virus (HBV)-positive sera and 10 hepatitis C virus (HCV)-positive sera.

HTLV-1 positive serum 13

HTLV-1 positive serum 15

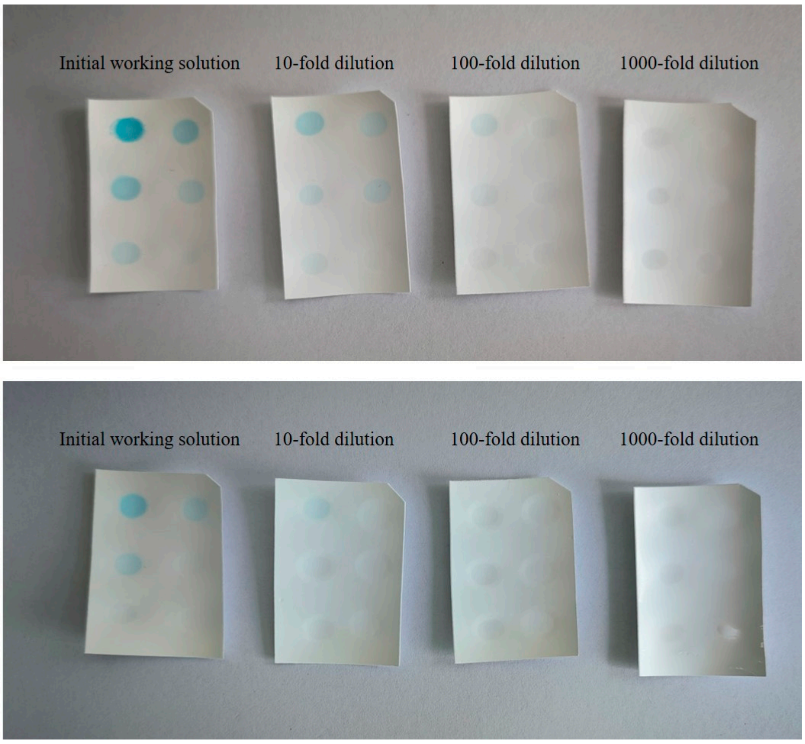

Figure S4. Dot-blot results for P19 and GP46 peptides using TMB detection for HLTV-1-postive sera 13 and 15 at four dilution levels (initial working solution, 10-fold dilution, 100-fold dilution, and 1000-fold dilution).
